# Implementation of the Fiala-based thermophysiological model coupled with the Zhang regression model of human thermal comfort

**DOI:** 10.1186/2046-7648-4-S1-A30

**Published:** 2015-09-14

**Authors:** Jan Pokorny, Miroslav Jicha

**Affiliations:** 1 Brno University of Technology, Energy Institute - Department of Thermodynamics and Environmental Engineering, Czech Republic

## Introduction

Evaluation of thermal comfort in non-uniform and transient environments is still a challenging topic, which requires knowledge of human physiology and perception of the thermal environment. We present the Matlab implementation of the Fiala thermophysiological model [1] coupled with the empirical model of human thermal comfort [2]. Both models are designed for non-uniform and transient environments, e.g. transport vehicle cabins. The original Fiala model was re-implemented with some modifications into commercial software as Theseus-FE or RadTherm and it has been widely used by the research community; e.g. to develop individualized models [3], [4], for coupling with the CFD (Computational Fluid Dynamics) [5] or for real-time applications [6]. Nowadays, [7] the Fiala model is one of the most advanced models in the field of the human thermophysiology and thermal comfort (index DTS - Dynamical Thermal Sensation).

## Methods

A mathematical background of the thermophysiological model is formed by the set of partial differential equations describing unsteady 1D heat transfer in living tissues. The set of equations was solved by the finite difference method using Crank-Nicolson scheme.

## Results

We implemented the Fiala-based model in the Matlab and we verified it on the experimental data from the literature. We also created a coupling between the Fiala and Zhang model although with only partial success.

## Discussion

The main problem of the coupling is the different definition of a neutral thermal state of the human body, which varies by 2 °C in the case of central body parts. This problem was pointed out and discussed by Schellen, et al. [8].

## Conclusion

The Fiala-based model was implemented into the Matlab and it was coupled with the Zhang regression model of thermal comfort. The prediction of the coupled models needs to be improved.
